# Exercise training as part of multidisciplinary rehabilitation in cancer survivors: an observational study on changes in physical performance and patient-reported outcomes

**DOI:** 10.1007/s00520-022-07351-5

**Published:** 2022-09-06

**Authors:** Anouk T. R. Weemaes, Matty P. Weijenberg, Antoine F. Lenssen, Milou Beelen

**Affiliations:** 1 Department of Physical Therapy, Maastricht University Medical Center+, Maastricht, the Netherlands; 2 Department of Epidemiology, Care and Public Health Research Institute (CAPHRI), Faculty of Health Medicine and Life Sciences, Maastricht University, Maastricht, the Netherlands; 3 Department of Epidemiology, School for Oncology and Reproduction (GROW), Faculty of Health Medicine and Life Sciences, Maastricht University, Maastricht, the Netherlands; 4 Department of Human Biology, School of Nutrition and Translational Research in Metabolism (NUTRIM), Faculty of Health Medicine and Life Sciences, Maastricht University, Maastricht, the Netherlands

**Keywords:** Oncology, Aerobic capacity, Muscle strength, Mental health, Fatigue, Quality of life

## Abstract

**Purpose:**

To describe changes in physical performance and patient-reported outcomes in cancer survivors who participated in an exercise program as part of usual-care multidisciplinary rehabilitation and the influence of training adaptations during the coronavirus-19 (COVID-19) pandemic.

**Methods:**

In an observational cohort study, cancer survivors underwent usual-care multidisciplinary rehabilitation including a 10-week exercise program. During the COVID-19 pandemic, the exercise program was adapted with reduced training time and frequency. Mean changes and 95% confidence intervals in physical performance (peak oxygen uptake (VO_2_peak), peak work rate during a steep ramp test (SRT-WRpeak), 6-min walking distance, muscle strength) and patient-reported outcomes (health-related quality of life, fatigue, anxiety, and depression) were assessed between the start and the end of the exercise program. Linear regression analysis, adjusting for baseline levels of outcomes, was used to investigate differences in changes in outcomes between participants who underwent the original and the adapted program.

**Results:**

All outcomes statistically significantly improved over time, regardless of adaptations in the exercise program. VO_2_peak increased with 9.6% and 7.7% in the original and adapted program, respectively. Significant smaller improvements were observed in SRT-WRpeak (− 3.9%) and upper body muscle strength (− 10.8%) after participation in the adapted compared to the original program. No significant between-group differences were observed for other outcomes.

**Conclusion:**

Physical performance and patient-reported outcomes statistically and clinically significantly improved in cancer survivors who participated in an exercise program as part of usual-care multidisciplinary rehabilitation. Improvements of performance outcomes were smaller since the training adaptations, though only significant for SRT-WRpeak and upper body strength.

## Introduction

Over the last decades, aging and improved diagnostics and treatment modalities have led to an increased number of cancer survivors. In 2018, approximately 50 million people worldwide were living with or beyond cancer [1]. Cancer survivors are often confronted with disease- and treatment-related side effects, like fatigue, declined aerobic capacity and muscle strength, anxiety and depression, and a diminished health-related quality of life (HRQoL) [2–6].

Research in the field of cancer survivorship care expanded in the past decades, and growing evidence emerged for the positive effects of exercise on the aforementioned side effects [7–10]. For this reason, international guidelines of the American College of Sports Medicine (ACSM) have emphasized the importance of the integration of exercise in cancer survivorship care [11].

While most studies focus on exercise interventions alone, multidisciplinary rehabilitation programs may better address the complex needs of patients with cancer [12]. Dutch cancer guidelines advocate prescription of a supervised, exercise-based multidisciplinary rehabilitation program to cancer survivors who experience combined physical and psychosocial problems [13]. Multidisciplinary rehabilitation commonly contains exercise training, supplemented by a range of treatments to improve mental health, chronic fatigue, work reintegration, body composition, and nutritional intake. Recently, two systematic reviews were published about the effects of multidisciplinary oncology rehabilitation in cancer survivors. Overall, rehabilitation resulted in positive effects on physical and psychosocial state, but the effects varied across studies [12, 14].

In recent exercise guidelines, the majority of available evidence on the efficacy of oncology rehabilitation is derived from randomized controlled trials (RCTs). RCTs have strengthened the body of proof for the efficacy of exercise in cancer rehabilitation, but have been reported to lack generalizability to the clinical setting [15, 16]. Patients have to meet pre-specified criteria (e.g., diagnosis, disease stage, age) in order to be eligible for enrollment in RCTs and have to give consent to participate. This might result in a healthier, fitter, and more motivated population, which may not be comparable to a broader population of cancer survivors [11]. While RCTs have the most powerful study design to investigate the efficacy of rehabilitation in a specific population under ideal circumstances, observational studies may be more appropriate to evaluate interventions in daily practice and in more heterogeneous populations with complex, chronic diseases such as cancer [15, 16].

In this observational study, we present data about physical performance and patient-reported outcomes in cancer survivors who participated in an exercise program as part of multidisciplinary rehabilitation between February 2019 and March 2021. Due to the coronavirus 2019 (COVID-19) pandemic, social distance policies and disruption of outpatient clinic care led to changes in the content of the training and a reduction in the training time and frequency. Therefore, this study had the following objectives:

The primary objective of this study was to describe changes in aerobic capacity, muscle strength, HRQoL, fatigue, and anxiety and depression in cancer survivors who participated in a 10-week exercise program as part of usual-care multidisciplinary rehabilitation.

The secondary objective was to compare changes in outcomes between the group of participants that followed the original program and the group of participants that followed an adapted exercise program, due to COVID-19 measures.

## Methods

### Participants

Participants were recruited from the multidisciplinary rehabilitation program at the Department of Physical Therapy of the Maastricht University Medical Centre (MUMC +) between February 2019 and March 2021. Patients participating in the program were asked for consent to use their exercise rehabilitation data. Patients who signed informed consent were included in the study. Participants were excluded if they were unable to follow the training program as intended. Patients were eligible for the program when they were ≥ 18 years, completed active medical treatment (i.e., surgery, chemotherapy, radiotherapy, stem cell transplantation) and were suffering from physical, and/or psychosocial complaints and/or chronic fatigue. Contraindications for participation in the rehabilitation program were the inability to perform basic activities of daily living and the presence of disabling comorbidities that seriously hamper physical exercise.

### Study design

This study was a prospective, longitudinal observational cohort study and all data were collected during usual-care multidisciplinary oncology rehabilitation at the MUMC + . Study procedures complied with the Declaration of Helsinki and were approved by the Medical Ethics Committee of the Maastricht UMC + with registration number 2018–0648.

### Rehabilitation program

Participants completed a 10-week supervised, group-based exercise program. The exercise program consisted of two training sessions weekly, both starting with 1 h of combined resistance and endurance training, followed by a 30-min break and, subsequently, 30 min of varying sports activities in the gym or swimming pool. In addition, participants took part in at least one of the following interventions: a psychoeducational intervention (seven individual or group-based sessions) guided by the psychologist or the social worker; fatigue management courses (six individual or group-based sessions) guided by the occupational therapist; return-to-work counselling (three individual sessions) guided by the occupational therapist; and dietary counselling (three individual sessions) delivered by the dietician. These additional programs were provided (partly) in parallel with the exercise program. Participants completed exercise tests and questionnaires before the start of the exercise program (T = 0) and in the week after completing the exercise program (T = 1). Of note is that some of the other interventions were not finished yet at T = 1.

### Measurements

#### Performance outcomes

A cardiopulmonary exercise test (CPET) was performed as part of usual-care, to screen for cardiopulmonary contraindications to exercise and to determine aerobic capacity. The CPET was conducted on a cycle ergometer (Lode Corival, Lode BV) as described previously [17]. After a warm-up phase, the work rate (WR) increased gradually according to an incremental ramp protocol, which was determined based on the participants’ self-reported physical activity level, aiming at a test duration of 8–12 min. The WR increased until the patient stopped cycling or pedaling frequency fell below 60 rpm. This point was defined as peak WR (CPET-WRpeak). Continuous breath-by-breath analysis was obtained using a spirometry system calibrated for respiratory gas and breathing volume measurements (Vyntus CPX, CareFusion Netherlands).

CPET results were analyzed by a trained researcher who was blinded for the moment of testing (T = 0 or T = 1), using a standardized protocol. Values of  oxygen uptake (VO_2_) and the respiratory exchange rate at WRpeak (VO_2_peak and RERpeak, respectively) were averaged over 30 s. An improvement in VO_2_peak of 1.0 mL/min/kg was found to be associated with a 9% risk reduction in all-cause mortality and was therefore considered a clinically relevant improvement [18]. Submaximal outcomes of CPET were determined as well. The VO_2_ at the ventilatory anaerobic threshold (VO_2_VAT) was determined using the “V-slope” method [19] and the ventilatory equivalents method [20]. The VO_2_ at the respiratory compensation point (VO_2_RCP) was determined using the ventilatory equivalents method and the minute ventilation/carbon dioxide production (VE/VCO_2_) slope [21]. The interrater reliability for determination of VO_2_VAT and VO_2_RCP was determined previously, when two researchers at Maastricht UMC + both analyzed 48 tests (partly from the current study) independently. This resulted in an intraclass correlation coefficient (ICC) of 0.95 (95% confidence interval (CI) 0.90–0.97) for determination of the VO_2_VAT and an ICC of 0.99 (95% CI 0.98–0.99) for determination of the VO_2_RCP, which indicates an excellent interrater reliability. The oxygen uptake efficiency slope (OUES) was derived from the relation between VO_2_ and minute ventilation, using the following formula: VO_2_ = (*a* × Log VE) + *b*, where *a* is the OUES [22].

In addition, patients performed a 6-min walk test (6MWT) and a steep ramp test (SRT). These tests also gave an indication of aerobic capacity and were performed as part of the usual-care rehabilitation program, to determine baseline training intensity. During the 6MWT, participants were instructed to walk as many meters as possible, on a marked 44-m course, within 6 min. Based on the minimal clinically important difference (MCID) of the 6MWT in adults with pathology, a change in the maximal walking distance of 30.5 m was considered clinically relevant [23]. The SRT was performed on a cycle ergometer (Lode Corival, Lode BV) as described previously [17]. After warming up, the WR increased with 25 W/10 s in a ramp-like manner, until the participant stopped cycling or pedaling frequency fell below 60 rpm. This point was defined as peak WR (SRT-WRpeak). The minimal detectable change in SRT-WRpeak was recently determined in survivors of cancer who participated in exercise-based multidisciplinary rehabilitation in MUMC +. Based on the findings of this study, an increase of 0.26 W/kg in SRT-WRpeak was seen as a true improvement [17].

Lower and upper body muscle strength was measured during submaximal repetition maximum (RM) tests on the leg press and chest press machine, respectively. The 5-RM was estimated for both exercises and the participant was asked to perform the maximum achievable number of repetitions up to five repetitions with this weight. When five repetitions were reached, the weight was increased and participants repeated the exercise after a 1-min pause until they no longer reached 5 repetitions. True 1-RM values were calculated afterwards using the Brzycki equation [24]. In March 2020, the gym at Maastricht UMC + was updated and the exercise machines were replaced by comparable new ones. Each participant performed strength tests at T = 0 and T = 1 on the same machines. A change in 1-RM chest press of 6.25 kg was considered clinically relevant, as determined in a study to the MCID of RM testing in patients with chronic obstructive pulmonary disease [25].

#### Patient-reported outcomes

HRQoL was measured using the European Organization for Research and Treatment of Cancer Quality of Life Questionnaire Core-30 (EORTC QLQ-C30). Each of the 30 item has to be rated on a scale from 1 to 4 and for two items from 1 to 7. This questionnaire distinguishes 15 subscales. The functioning scales (physical, role, emotional, social, and cognitive functioning), the global QoL scale, and a functioning sum score (averaged across the 15 items that belong to the functioning scales) were calculated. Sub-scores as well as sum scores were linearly transformed on a 100-point scale, with higher scores indicating higher levels of HRQoL [26, 27]. A change of 10 points on each subscale or the sum score was considered clinically relevant [28, 29].

Fatigue was assessed using the Multidimensional Fatigue Inventory-20 (MFI-20), which is a 20-item questionnaire with a five-dimensional structure (general, physical and mental fatigue, reduced motivation and activity). Each item is scored on a 5-point Likert scale. The sub-scores range from 4 to 20, with lower scores indicating lower levels of fatigue. The sum score was calculated by adding up the sub-scores [30]. Changes on the MFI-20 subscales that exceeded MCIDs as determined in a cohort of patients with cancer receiving radiotherapy (ranging from 3.18 to 3.80 for different subscales) were considered clinically relevant [31].

Anxiety and depression were assessed using the 14-item Hospital Anxiety and Depression Scale (HADS). Items are scored on a 4-point scale and sub-scores for anxiety and for depression range from 0 to 21, with lower scores indicating lower levels of anxiety and depression. The sum score was calculated by adding up the sub-scores [32]. A change of 1.7 points for each sub-score was considered clinically relevant, as assessed in patients with cardiovascular disease in the study of Lemay et al. [33].

### Other measurements

Age, cancer type, presence of metastasis and comorbidities, treatment type, and time since treatment at T = 0 were extracted from medical records. Height and weight were measured at T = 0 and T = 1, after which body mass index (BMI) was calculated. The training compliance (%) was calculated by dividing the number of training sessions that participants attended, by the number of planned training sessions, multiplied by 100. Indication for other interventions in the rehabilitation program and completion rates of these therapies at T = 1 were reported as well.

### Exercise protocol

Participants performed four strengthening exercises each session, targeting large muscle groups of the upper and lower body, and core. Resistance training consisted of three sets of 8–12 repetitions and training intensity was set at 60% of the participant’s initial 1-RM. Endurance training in the first training session of the week consisted of 20-min walking on a treadmill, with a walking speed of 80% of their mean speed in the baseline 6-MWT. In the other training session, participants performed two sets of 10 min of interval training on a cycle ergometer, one set before and one after the resistance training program. Intervals were performed for 30 and 60 s at 65% and 30% of the participant’s SRT-WRpeak, respectively [34]. The training load was adjusted in a personalized manner, according to the 0–10 Borg rating of perceived exertion and weekly increase in load was aimed for in order to reach overload. A moderate- to high-intensity exercise was pursued for all training components, corresponding with a Borg score of 4–6 [35].

### COVID-19

The rehabilitation program was interrupted between March 2020 and July 2020, because all outpatient activities were cancelled in that time frame, due to COVID-19 measures. Rehabilitation data of participants who were enrolled in the exercise program prior to this period and did not finish yet were excluded from this study because measurements at T = 1 were cancelled or postponed. In July 2020, national guidelines permitted resumption of the rehabilitation program. Because of the social distancing policies, exercise training took place in smaller groups of four instead of eight patients. In order to avoid a long waiting list, the training frequency was reduced to once weekly. Because there was only one training session weekly, endurance training was changed to 10-min walking and 10-min cycling in one session. Intensity of the endurance training remained the same. Contact sports and swimming were not allowed, so patients could only perform the endurance and resistance training program. For participants who were recruited from July 2020 onwards, the frequency, time, and type of exercise training changed. However, we encouraged all participants to be physically active on other weekdays and to perform body weight strengthening exercises at home once to twice weekly. Online instructions for a home-based program with strengthening exercises were offered to all participants. Other interventions of the rehabilitation program took place in smaller groups as well, or via phone calls.

### Statistical analysis

Continuous variables were checked for normality using histograms and Q-Q plots and are presented as mean ± SD or median and interquartile ranges, as appropriate. Categorical variables are presented as frequencies (*n*) and percentages (%). Performance- and patient-reported outcomes are reported for the group of participants that completed the original exercise program and the participants who completed the adapted program since COVID-19. Outcomes are reported for measurements at T = 0 and at T = 1. Mean changes between T = 0 and T = 1 in outcome variables within individuals are reported with 95% CI. Mean differences (and 95% CI) in change scores between participants who underwent the original program and the adapted program were estimated using linear regression analysis, with the change scores as outcome, a group variable (indicating whether individuals followed the original or adapted exercise program) as dependent variable, and additional adjustment for the absolute values of the outcomes at T = 0. Differences between changes in muscle strength were reported as percentages as well, to account for possible differences in baseline values due to the change of the exercise machine during the study. If the confidence intervals did not include zero, the mean change or difference in change was considered statistically significant. MCIDs are reported when they were available in literature. Changes were considered clinically relevant when they exceeded MCIDs. Statistical analyses were performed using SPSS version 25.0 (SPSS Statistics for Windows, version 25.0, IBM Corp.).

## Results

### Participants

**Fig. 1:**
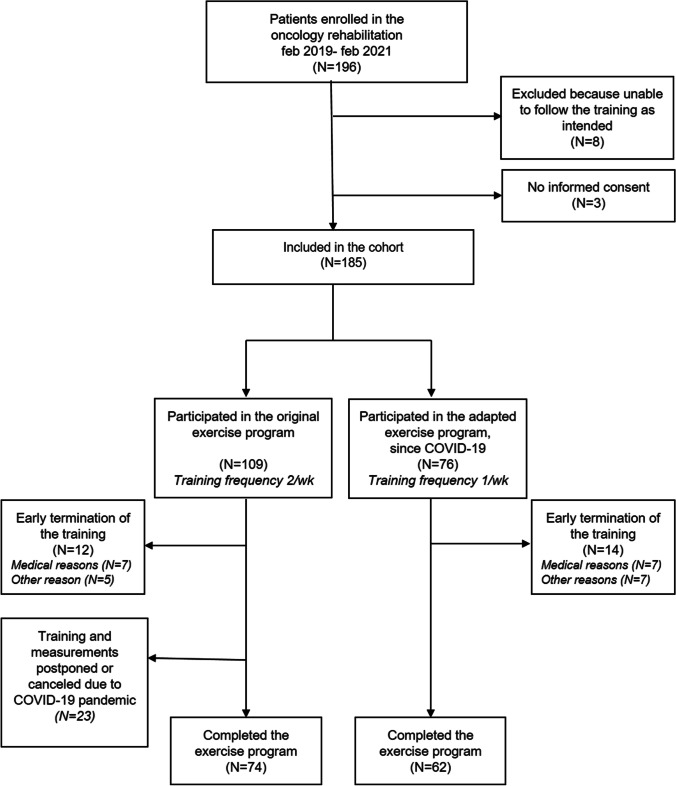
Participant flowchart

A total of 196 patients participated in the multidisciplinary oncology rehabilitation program at MUMC + between February 2019 and March 2021.Three of them gave no informed consent for the use of their data resulting in a participation rate of 98.4%. Eight participants were excluded because they were unable to follow the exercise training as intended (i.e., because of physical impairments, absence for longer periods). This resulted in a final sample size of 185 subjects. Seventy-four and 62 participants completed the original exercise program and the adapted program since the COVID-19 pandemic, respectively. Twelve (11.0%) and 14 (18.4%) participants were unable to complete the original and the adapted exercise program due to medical or other reasons, respectively. Twenty-three out of 109 (21.1%) participants were lost to follow-up due to COVID-19 (Fig. 1).

Of the total population, 143 participants (77.3%) were women. Mean age was 55.7 ± 11.5 years and mean BMI was 27.9 ± 5.3 kg/m^2^. Breast cancer was the most common diagnosis (46.5%). Subjects started with the exercise program on average 4.7 ± 4.4 months after completing active medical treatment. Patient characteristics were not significantly different between groups (Table 1).

**Table 1:** Characteristics of cancer survivors who started multidisciplinary rehabilitation overall and according to starting the rehabilitation before or after the start of the COVID-19 pandemic

	*Total population of cancer survivors* *N* = *185*	Cancer survivors who participated in the original exercise program (Training frequency 2/wk) *N* = *109*	Cancer survivors who participated in the adapted exercise program, since the COVID-19 pandemic (Training frequency 1/wk) *N* = *76*
Sex (*n*, %)			
Male	42 (22.7)	27 (24.8)	15 (19.7)
Female	143 (77.3)	82 (75.2)	61 (80.3)
Age (years)	55.7 ± 11.5	56.2 ± 11.0	54.9 ± 12.2
Body height (cm)	169.0 ± 7.9	169.2 ± 8.0	168.6 ± 7.9
Body mass (kg)	79.3 ± 14.4	79.0 ± 13.8	79.8 ± 15.3
Body mass index (kg/m^2^)	27.9 ± 5.3	27.6 ± 5.0	28.2 ± 5.8
Cancer type (*n*, %)			
Breast cancer	86 (46.5)	51 (46.8)	35 (46.1)
Lung cancer	15 (8.1)	10 (9.2)	4 (5.3)
Colorectal cancer	14 (7.6)	8 (7.3)	7 (9.2)
Lymphomas	9 (4.9)	5 (4.6)	4 (5.3)
Leukemia	9 (4.9)	3 (2.8)	6 (7.9)
Cervix	9 (4.9)	5 (4.6)	4 (5.3)
Prostate	7 (3.8)	4 (3.9)	3 (3.7)
Other	36 (19.5)	27 (24.8)	16 (21.1)
Metastasis (*n*, %)			
Lymphatic metastasis	38 (20.5)	18 (16.5)	20 (26.3)
Distant metastasis	16 (8.6)	11 (10.1)	5 (6.6)
No metastasis	131 (70.8)	80 (73.4)	51(67.1)
Treatment (*n*, %) ^a^			
Surgery	138 (74.6)	82 (75.2))	56 (73.7)
Chemotherapy	121 (65.4)	65 (59.6)	56 (73.7)
Radiotherapy	100 (54.1)	47 (43.1)	38 (50.0)
Hormone therapy	57 (30.8)	33 (30.3)	24 31.5)
Immunotherapy	25 (13.5)	9 (8.3)	16 (21.1)
Stem cell transplantation	8 (4.3)	3 (2.8)	5 (6.6%)
Time since treatment (months)	4.7 ± 4.4	4.9 ± 4.9	4.4 ± 3.6
Comorbidity (*n*, %) ^a^			
Cardiovascular	45 (24.3)	20 (26.3)	25 (22.9)
Respiratory	15 (8.1)	8 (7.3)	7 (9.2)
Diabetes	8 (4.3)	6 (5.5)	2 (2.6)

Values are presented as *n* (%) for categorical variables and as mean ± SD for continuous variables

^a^Sums of percentages are higher than 100% because participants received more than one type of treatment

^b^Sums of percentages are less than 100% because not all participants were suffering from comorbidities

### Rehabilitation program

The training compliance rate was 93.7 ± 7.7% and 91.37 ± 11.8% in participants who completed the original and the adapted program, respectively. The percentage of indication for other interventions and their completion rates at T = 1 did not differ notably between the groups, but the other interventions were often not completed yet at T = 1 (Table 2).

**Table 2:** Participation of cancer survivors undergoing multidisciplinary rehabilitation in other interventions than the exercise intervention

Other interventions (*n*, %) indicated / *completed*	Cancer survivors who participated in the original exercise program (training frequency 2/wk) *N* = *74*	Cancer survivors who participated in the adapted exercise program, since the COVID-19 pandemic (training frequency 1/wk) *N* = *62*
Psychology	58 (78.4)	50 (80.6)
*Completed module at T* = *1*^*a*^	*14 (24.1)*	*11 (22.9)*
Occupational therapy fatigue	57 (77.0)	42 (67.8)
*Completed module at T* = *1*^*a*^	*8 (14.0)*	*7 (16.7)*
Occupational therapy return to work	36 (48.7)	32 (51.8)
*Completed module at T* = *1*^*a*^	*5 (13.9)*	*4 (12.5)*
Dietetics	12 (16.2)	10 (16.1)
*Completed module at T* = *1*^*a*^	*8 (66.7)*	*2 (0.2)*

Data is presented only for participants that completed the exercise training

The frequency and percentage (*n*, %) of participants that were indicated for an intervention are presented. Of these participants that were indicated for the module, the frequency and percentage (*n*, %) of participants that completed the module at the end of the exercise program (T = 1) are presented

^a^Note that this was the status of completion of the intervention at T = 1 and that in many instances the interventions were still ongoing

2/wk, twice weekly; 1/wk, once weekly

### Changes in physical performance and patient-reported outcomes

All measures of aerobic capacity and muscle strength improved statistically and clinically significantly after 10 weeks of exercise training in both groups. An increase of 1.9 mL/kg/min (9.6%) and 1.4 mL/kg/min (7.2%) in VO_2_peak was observed after participation in the original and the adapted program, respectively (Table 3). Patient-reported outcomes for HRQoL, fatigue, and anxiety and depression improved statistically significantly after 10 weeks of exercise training, both before and after the changes in the program due to COVID-19. Clinically relevant improvements in HRQoL were reached in four out of six subscales of the EORTC-QLQ-C30 in the original program and five out of six subscales in the adapted program. A clinically relevant decrease in general and physical fatigue on the MFI was observed for both groups. Clinical relevant improvements on the HADS were seen only in the depression subscale before the adaptations in the program and in the anxiety and the depression scale after the adaptations (Table 4).

**Table 3:** Changes in mean outcomes of performance tests within groups and differences in changes in outcomes between the group of cancer survivors that participated in the original exercise program as part of multidisciplinary rehabilitation and the group of cancer survivors that participated in the adapted exercise program since COVID-19

		Cancer survivors who participated in the original exercise program (training frequency 2/wk)				Cancer survivors who participated in the adapted exercise program, since the COVID-19 pandemic (training frequency 1/wk)				Difference between groups (*adapted program- original program*) corrected for baseline values	
	MCID ^a^	T = 0	T = 1	Change absolute | %	95% CI	T = 0	T = 1	Change absolute | %	95% CI	Difference	95% CI
Aerobic capacity		***N* = *65***				***N* = *57***					
CPET											
VO_2_peak (mL/kg/min) [18]	1.0	19.8 ± 5.2	21.7 ± 6.2	1.9 † | 9.6%	1.3–2.5*	19.5 ± 6.0	20.9 ± 5.9	1.4 † | 7.2%	0.8–1.9*	− 0.5	− 0.3 to 1.3
CPET WRpeak (W/kg)	N.A	1.7 ± 0.6	1.9 ± 0.7	0.2 | 11.8%	0.1–0.2*	1.6 ± 0.6	1.7 ± 0.7	0.1 | 6.3%	0.1–0.2*	− 0.1	− 0.1 to 0.0
*RERpeak*		*1.16* ± *0.10*	*1.18* ± *0.10*			*1.15* ± *0.10*	*1.15* ± *0.11*				
VO_2_ AT (mL/kg/min)	N.A	11.7 ± 2.5	13.3 ± 3.3	1.6 | 13.7%	1.1–2.2*	12.1 ± 3.0	13.8 ± 3.4	1.6 | 13.2%	1.1–2.2*	0.0	− 0.8 to 0.8
OUES (/kg)	N.A	22.7 ± 5.4	24.1 ± 6.1	1.4 | 6.2%	0.7–2.1*	22.5 ± 6.6	23.6 ± 6.3	1.2 | 5.3%	0.4–1.9*	− 0.3	− 1.3 to 0.7
RCP reached ^b^		48 (44.4)				36 (46.8***)***					
VO_2_ RCP (mL/kg/min)	N.A	18.7 ± 4.3	20.8 ± 4.9	2.1 | 11.2%	1.5–2.7*	19.0 ± 5.4	20.5 ± 5.6	1.4 | 7.4%	0.7–2.2*	− 0.7	− 1.6 to 0.3
SRT WRpeak (W/kg) [17]		***N* = *63***				***N* = *55***					
	0.26	3.12 ± 0.92	3.48 ± 0.96	0.36 † | 11.5%	0.28–0.44*	3.02 ± 0.88	3.25 ± 0.88	0.23 | 7.6%	0.14–0.32*	− 0.13	− 0.25 to − 0.02*
6-MWD (m) [23]		***N* = *71***				***N* = *58***					
	30.5	514 ± 104	577 ± 101	63 † | 12.3%	51–75*	515 ± 92	564 ± 104	49 † | 9.5%	36–63*	− 14	− 31 to 4
Muscle strength ^c^		***N* = *71***				***N* = *55***					
1- RM leg press (kg)	N.A	86.3 ± 22.8	118.6 ± 35.1	32.3 | 37.4%	26.6–38.0*	108.2 ± 29.2	137.0 ± 33.8	28.8 | 26.6%	22.8–34.9*	− 3.2 |− 10.8%	− 12.3 to 5.8 |− 2.9% – 20.4%
		***N* = *67***				***N* = *54***					
1-RM chest press (kg) [25]	6.25	24.1 ± 11.3	35.8 ± 14.6	11.7 † | 48.5%	9.7 – 13.7*	27.0 ± 11.2	32.7 ± 11.8	5.7 | 21.5%	3.8 – 7.7*	− 6.0 |− 27.0%	− 8.7 to − 3.0* |− 50.2% to − 9.2%*

Means ± SD were presented for both groups and changes within groups were calculated with corresponding 95% confidence intervals (CI). *Statistically significant, ^†^Clinically relevant

Changes in outcomes were compared between both groups. Differences in change scores between groups were reported in the last column with 95% CI, corrected for baseline values, which were calculated using linear regression

^a^Minimally clinically important differences are provided when they have been determined and reported in previous studies, as described in the “Methods” section. References are provided in the first column of the table []. When MCIDs were not available in literature, this was reported as not available (N.A.)

^b^Frequencies and percentages of participants that reached RCP at both tests (T = 0 and T = 1) were reported, with corresponding values

^c^Differences in change scores between groups, with corresponding 95% CI, are given in absolute numbers and | percentages, to account for baseline differences due to the usage of different exercise machines

*CPET* cardiopulmonary exercise test, *VO*_*2*_*peak* peak oxygen uptake, *WRpeak* peak work rate, *RERpeak* peak expiratory exchange rate, *VO*_*2*_* AT* oxygen uptake at anaerobic threshold, *VO*_*2*_* RCP* oxygen uptake at respiratory compensation point, *OUES* oxygen uptake efficiency slope, *SRT* steep ramp test, *6-MWT* 6-min walking test, *1-RM* one-repetition maximum. *2/wk* twice weekly, *1/wk* once weekly, *MCID* minimally clinically important difference, *N.A.* data not available

**Table 4:** Changes in mean outcomes of patient-reported outcomes within groups and differences in changes in outcomes between the group of cancer survivors that participated in the original exercise program as part of multidisciplinary rehabilitation and the group of cancer survivors that participated in the adapted exercise program since COVID-19

	MCID	Cancer survivors who participated in the original exercise program (training frequency 2/wk)				Cancer survivors who participated in the adapted exercise program, since the COVID-19 pandemic (training frequency 1/wk)				Difference between groups (*adapted program − original program*) corrected for baseline values	
		T = 0	T = 1	Change	95% CI	T = 0	T = 1	Change	95% CI	Difference	95% CI
Quality of life	MCID ^a^	***N* = 62**				***N* = 47**					
(EORTC-QLQ-C30) [28, 29]											
Global QoL	10.0	56.86 ± 16.15	71.10 ± 16.09	14.24 †	9.7–18.8*	57.45 ± 16.23	68.79 ± 18.76	11.34 †	6.6–16.1*	− 2.84	− 8.63 to 2.95
Physical functioning	10.0	72.04 ± 15.59	83.83 ± 12.26	11.40 †	8.2–14.6 *	70.33 ± 18.67	83.83 ± 12.26	13.50 †	8.8–18.2*	1.45	− 2.84 to 5.74
Role functioning	10.0	55.28 ± 25.00	70.43 ± 20.56	15.16 †	9.4–20.9*	52.13 ± 26.83	74.82 ± 22.75	22.70 †	15.5–29.9*	5.42	− 1.57 to 12.41
Emotional functioning	10.0	63.98 ± 26.69	73.39 ± 23.80	9.40	3.7–15.0 *	61.35 ± 21.73	78.55 ± 21.73	17.20 †	11.0–23.4*	6.42	− 13.18 to 0.35
Cognitive functioning	10.0	62.26 ± 28.30	69.08 ± 25.93	6.80	0.9–12.7*	62.41 ± 28.12	72.34 ± 23.90	9.93	3.7–16.2*	3.17	− 4.14 to 10.49
Social functioning	10.0	59.41 ± 24.82	79.04 ± 26.98	19.63 †	12.7–26.5*	62.06 ± 29.01	78.72 ± 22.44	16.67 †	10.1–23.3*	− 1.62	− 9.88 to 6.64
Sum score functioning	10.0	62.60 ± 17.86	75.07 ± 17.86	12.48 †	8.5–16.5*	61.65 ± 18.51	77.65 ± 14.84	16.00 †	12.2–19.8 *	3.14	− 1.76 to 8.03
Fatigue (MFI) [31]		***N* = 63**				***N* = 43**					
General fatigue	3.18	16 ± 3	12 ± 4	− 3 †	− 4 to − 3*	16 ± 3	12. ± 4	− 4 †	− 5 to − 3 *	0	− 2 to 1
Physical fatigue	3.45	16 ± 3	10 ± 4	− 6 †	− 7 to − 4*	15 ± 4	10 ± 5	− 5 †	− 7 to − 4*	0	− 1 to 2
Reduced motivation	3.60	11 ± 4	9 ± 4	− 2	− 3 to − 2*	12 ± 4	9 ± 4	− 3	− 4 to − 2*	0	− 1 to 1
Reduced activity	3.50	14 ± 4	11 ± 4	− 3	− 4 to − 2*	14 ± 4	11 ± 4	− 4 †	− 5 to − 3*	0	− 2 to 1
Mental fatigue	3.80	13 ± 4	12 ± 5	− 1	− 2 to 0*	13 ± 5	12 ± 5	− 1	− 3 to 0*	0	− 2 to 1
Sum score		69 ± 15	54 ± 17	− 15	− 19 to − 12*	71 ± 12	54 ± 17	− 17	− 22 to − 12*	− 1	− 7 to 4
Anxiety and depression (HADS) [33]		***N* = 59**				***N* = 39**					
Anxiety	1.7	8 ± 5	7 ± 4	− 1	− 2 to 0*	9 ± 4	7 ± 4	− 2 †	− 3 to − 1*	0	− 2 to 1
Depression	1.7	7 ± 5	5 ± 4	− 2 †	− 3 to − 1*	7 ± 4	5 ± 5	− 2 †	− 3 to − 1*	0	− 1 to 2
Sum score	N/A	15 ± 9	12 ± 8	− 4	− 5 to − 2*	16 ± 7	12 ± 8	− 4	− 6 to − 2*	0	− 2 to 2

Means ± SD were presented for both groups and changes within groups, with corresponding 95% confidence intervals (CI). *Statistically significant, ^†^Clinically relevant

Changes in outcomes were compared between both groups. Differences in change scores between groups were reported in the last column with 95% CI, corrected for baseline values, which were calculated using linear regression. *EORTC-QLQ-C30* European Organization for Research and Treatment for Cancer Quality of Life Questionnaire, *MFI* Multidimensional Fatigue Inventory, *HADS* Hospital Anxiety and Depression Score, *2/wk* twice weekly, *1/wk* once weekly

^a^Minimally clinically important differences are provided when they have been determined and reported in previous studies, as described in the “Methods” section. References are provided in the first column of the table []

### The influence of training adaptations

For nearly all performance outcomes, changes over time were more pronounced before the adaptations in the program, albeit only statistically significantly different for SRT-WRpeak and upper body strength. Mean upper body strength improved with 48.5% in participants who took part in the original program and with 21.5% in participants who took part in the adapted program. Mean improvement in SRT-WRpeak improved with 0.36 W/kg (CI 0.28–0.44) or 11.5% in participants in the original program and 0.23 (CI 0.14–0.32) W/kg or 7.6% in participants in the adapted program since COVID-19 (Table 3). In contrast with results of the performance tests, improvements in patient-reported outcomes were not different between the groups that participated before or since training adaptations (Table 4).

## Discussion

The results of this study showed significant improvements in aerobic capacity, muscle strength, HRQoL, fatigue, anxiety, and depression in cancer survivors following a 10-week exercise program as part of usual-care multidisciplinary oncology rehabilitation. Changes were clinically relevant for nearly all outcomes; MCIDs were not available in literature for 1-RM leg press, submaximal outcomes of CPET, and for the sum score of the HADS. For SRT-WRpeak, only the minimal detectable change was available, which was therefore used to compare our study results with. A significant and clinically relevant improvement in VO_2_peak of 1.9 mL/min/kg and 1.4 mL/min/kg was seen after participation in the original program and the adapted program since COVID-19, respectively. In a meta-analysis by Scott et al. on the effects of exercise therapy on aerobic capacity in cancer survivors, a larger improvement of 2.8 mL/min/kg (CI weighted mean difference 1.58–2.67 mL/min/kg) in mean VO_2_peak was observed [9]. Current guidelines of ACSM prescribe an 8–12-week combined aerobic and resistance exercise program with moderate intensity three times weekly to improve physical function [11]. The lower improvements in VO_2_peak found in the current study might be explained by a lower training frequency. Moreover, the moderate- to high-intensity training prescribed in the current study was potentially not always reached due to limited adherence of training intensity. Finally, the training intensity in the current study was based on baseline performance tests and perceived exertion. Training frequency, type, and time were equal for all participants and could be more personalized in the future.

Another plausible explanation for this inconsistency is the fact that Scott et al. only included RCTs in the meta analyses, which might have resulted in a fitter population [15, 16]. Besides, this meta-analysis focused on the effects of exercise alone, while the current study investigated the effects of exercise as part of multidisciplinary rehabilitation, in patients with more complex care needs. Surprisingly, a systematic review of Dennett et al. showed no significant effects for supervised exercise-based, multidisciplinary rehabilitation on VO_2_peak, which was attributed to issues with exercise prescriptions, which are often not well-reported in trials [14]. In both reviews, a large heterogeneity between studies was seen.

In our study, a significant improvement in HRQoL was seen after participation in the original program (sum score EORTC-QLQ-C30 + 12.48) and the adapted program (sum score EORTC-QLQ-C30 + 16.00). Comparable improvements were seen in a study on the effectiveness of a 12-week, multidisciplinary rehabilitation program in breast cancer patients (sum score EORTC-QLQ-C30 + 11.67) [36]. However, since most of our participants did not yet complete the other interventions at T = 1, improvements in patient-reported outcomes probably could have been larger. Studies on oncology rehabilitation vary a lot in content, duration, and timing of the programs and in reported outcome measures. Therefore, more extensive comparison of our study results with existing literature was not possible.

In this study, we also compared changes in outcomes between participants who exercised twice weekly in the original program and participants who exercised once weekly due to changes in the program since the COVID-19 pandemic. Significant between-group differences were observed for SRT-WRpeak and upper body muscle strength, with larger improvements for the group that participated in the original program. This is not surprising, because participants in this group attended the exercise training twice weekly. When looking at changes in the other performance outcomes, non-significant differences were seen between groups, with larger improvements for the group in the original program. Attention for habitual physical activity guidelines may have increased when training frequency and time were diminished since COVID-19. Consequently, participants may have been more active outside the training program since the training adaptations, which could have reduced the expected difference in training improvements. No significant between-group differences were seen for patient-reported outcomes. Unexpectedly, improvements in HRQoL did not decrease under the circumstances of the COVID-19 pandemic (e.g., social isolation, anxiety). This could have been due to a “response shift”, referring to changes in internal standards and values during a crisis [37]. This study was not originally designed to investigate the differences between groups; therefore, caution is warranted when drawing conclusions from this study based on significance testing alone.

A strength of this study was the observational design and the fact that data was collected during daily practice. The results might give a more realistic reflection of the physical and psychosocial changes after an oncology rehabilitation program, when compared to RCTs with strict inclusion criteria, in an experimental setting [15, 16]. Another strength was the fact that this study investigated a multidisciplinary rehabilitation program, which is best suited in this population with complex care needs, but has not often been studied before. Furthermore, data on different outcome measures were collected, covering not only physical but also psychosocial issues and fatigue. The observational design was not only a strength, but also a limitation of this study, because it is more challenging to draw firm conclusions about the changes in outcomes without a control group and random group assignment. It is likely that the natural course of improvement in physical performance and patient-reported outcomes after cancer treatment has played a role in the observed changes in outcomes over time. However, in the meta-analysis of Scott et al., a negligible mean improvement in VO_2_peak of 0.2 mL/kg/min was seen in patients with cancer who received no exercise intervention. The fact that participants took part in interventions other than the exercise training during this study could be seen as a limitation as well. Although patient-reported outcomes may have been influenced by other interventions than the exercise interventions alone (e.g., psychoeducational intervention, fatigue- and return-to-work counseling), these interventions were less likely to have influenced performance outcomes since they did not contain exercise elements. Further of note is that this study was aimed at investigating cancer patients with both physical and psychosocial complaints and/or chronic fatigue. Therefore, the findings of the current study cannot be generalized to all cancer survivors.

## Conclusion

The results of this study indicate that cancer survivors with both physical and psychosocial complaints significantly improve in aerobic capacity, muscle strength, HRQoL, fatigue, anxiety, and depression during a 10-week supervised, group-based exercise program as part of usual-care multidisciplinary oncology rehabilitation. Reductions in frequency, time, and type of training during the COVID-19 pandemic still resulted in significant improvements of all outcomes. However, improvements of most performance outcomes appeared to be smaller since the training adaptations, though only significant for SRT-WRpeak and upper body strength.

## Data Availability

Study data are available on request from the corresponding author.
